# Supplementary material for: A scalable gut epithelial organoid model reveals the genome-wide colonization landscape of a human-adapted pathogen
Source: Nat Genet. 2025 Jun 12;57(7):1730–41. doi: 10.1038/s41588-025-02218-x (PMC12283395; doi:10.1038/s41588-025-02218-x)
Supplement: Supplementary file 1 — Supplementary Notes 1–3. [file 41588_2025_2218_MOESM1_ESM.pdf]

nature genetics

Supplementary information

https://doi.org/10.1038/s41588-025-02218-x

A scalable gut epithelial organoid model reveals the genome-wide colonization landscape of a human-adapted pathogen

In the format provided by the authors and unedited

Supplementary Informations

TABLE OF CONTENT

Supplementary Note 1. ZINB model for TraDIS screen……………………………………………2

Supplementary Note 2. Caco-2 cell culture and infections……………………………………….5

Supplementary Note 3. Bacterial RNA extraction……………………………………………….6

References………………………………………………………………………………………..6

SUPPLEMENTARY NOTES

Supplementary Note 1. ZINB model for TraDIS screen.

Full source code was deposited to Zenodo at the following link: https://doi.org/10.5281/zenodo.15096674[1].

Definition of variables and parameters

$Y_{rgk,\text{in/out}}$: input/output read count of insertion site k in gene g and replicate r.

N_g: number of genes

$N_{k,r}$: number of non-zero insertion sites in the input sample of replicate r.

$N_{r,\text{in/out}}$: total number of read counts in the input/output of replicate r.

$N_r = \frac{N_{r,\text{out}}}{N_{r,\text{in}}}$: ratio between sum of output and sum of input counts in replicate r.

n_r: normalization factor between input and output sample of replicate r.

$\alpha_{rgk,\text{in}} = log(Y_{rgk,\text{in}})$: input log-counts, strictly positive, because insertion sites have been removed from the replicates where they have a zero-count in the input sample.

$\overline{\alpha}_{r,\text{in}} = \frac{1}{N_{k,r}} \sum_{g,k} \alpha_{rgk,\text{in}}$: mean of input log-counts in replicate r.

$\tilde{\alpha}_{rgk,\text{in}} = \alpha_{rgk,\text{in}} - \overline{\alpha}_{r,\text{in}}$: log-count normalized by mean count of replicate r.

$\alpha_{r,\text{max}} = \max(\tilde{\alpha}_{rgk,\text{in}})$: maximum normalized log-count of replicate r.

Model description. We developed a Bayesian model to extract genome-wide gene-wise fitness scores in the presence of experimental bottlenecks (Fig. 2d, Extended Data Fig. 3f). The processed input counts align well with a negative binomial (NB) distribution (Extended Data Fig. 3h), a standard choice for modeling count data from next-generation sequencing technologies. However, the infection bottleneck introduces zero-inflation in the output data (Extended Data Fig. 3h), rendering the NB distribution inadequate for capturing the probability of observing a zero-count in the output libraries. Consequently, we employed a zero-inflated negative binomial (ZINB) model, constituting a mixture of an NB model and an additional technical "zero" component.

Given the unique mutant composition of individual replicates (Fig. 2d, Extended Data Fig. 3g), we encountered challenges in modeling gene-wise mean counts. Instead, we assumed that the output counts for a single insertion site $Y_{rgk,\text{out}}$ follow a ZINB distribution, where their probability of a stochastic (technical) zero due to experimental bottlenecks is given by the mixing coefficient θ_{rgk}. The mean of the NB distribution depends on the normalized read count of the insertion site in the corresponding input sample and the gene-wise log-fold change logFC_g between input and output abundance of all insertion sites in gene g across all replicates:

$$ZINB(Y_{rgk,\text{out}}; \mu_{rgk}, \phi_{rgk}) = \begin{cases} \theta_{rgk} + (1-\theta_{rgk})NB(0; 0, \phi_{rgk}), \text{ if } Y_{rgk,out} = 0 \\ (1-\theta_{rgk})NB(Y_{rgk,out}; \mu_{rgk}, \phi_{rgk}), \text{ if } Y_{rgk,out} > 0 \end{cases}, \quad (1)$$

where $\mu_{rgk} = exp\left(\alpha_{rgk,\text{in}} + n_r + \text{logFC}_g\right)$ denotes the expected output count. The model parameter logFC_g uses the natural logarithm.

The technical zero probability θ_{rgk} for the insertion site k in gene g and replicate r depends on the fraction of zero counts in the output library compared to the input library $f_{z,r}$ (Extended Data Fig. 3i) and the log-count in the input $\alpha_{rgk,\text{in}}$ (Extended Data Fig. 3j). We assumed the abundance-dependence to be linear and utilized a logit linker function, i.e.

$$\theta_{rgk} = \text{logit}^{-1}\left(f_{z,r} + a_1 + a_2 \cdot \alpha_{rgk,\text{in}}\right),$$

where a_1 and a_2 are model parameters.

Furthermore, we assumed that highly abundant insertion sites are measured more accurately, which we account for by making the dispersion coefficient of the NB model gene- and abundance-dependent

$$\phi_{rgk} = \phi_g \cdot \left(1 + b \cdot \frac{\tilde{\alpha}_{rgk,\text{in}}}{\alpha_{r,\text{max}}}\right),$$

where $b \in [-1,1]$ to ensure that the gene-wise dispersion is multiplied by a factor between 0 and 1. By integrating the technical zero probability into the mixing coefficient, zeros due to a fitness defect contributes to the gene-wise log-fold change, which represent the differential abundance in the output samples corrected for technical loss.

Normalization and modeling of infection bottlenecks. To correct for sequencing depth and infection bottlenecks, we extracted the normalization factors n_r and the mixing coefficients θ_{rgk} from the 12,573 insertion sites located within pseudogenes. Given that pseudogenes are non-functional, mutations in these regions should not confer fitness advantages or defects, i.e.

$$\text{logFC}_g = 0.$$

With a minimum of 145 insertion sites in pseudogenes per replicate, pseudogenes provide a sufficient basis for determining the normalization factor across all replicates.

As insertion sites are unique to each input-output pair, pairwise normalization factors n_r suffice. We corrected the normalization factor for differences in library size between input and output

$$n_r = log(N_r) + \tilde{n}_r,$$

where $\tilde{n}_r$ is a model parameter, representing a global log-fold change between input and output counts of replicate r.

Furthermore, this step involves capturing the dependence of the mixing coefficient θ_{rgk} on the log-abundance of the insertion site in the input sample $\alpha_{rgk,\text{in}}$ and the fraction of insertion sites with zero-count in the output $f_{z,r}$, as well as the abundance dependence of the dispersion ϕ_{rgk}. Thus, we fit the parameters a_1, a_2 and b as defined above.

To improve the variance and normalization modeling by sharing information across genetic loci and sequencing libraries, we imposed a hierarchical structure:

$$\phi_g \sim N(\mu_\phi, \sigma_\phi) \text{ and } \tilde{n}_r \sim N(\mu_n, \sigma_n).$$

The remaining model parameters were assigned either Gaussian (N) or Cauchy (C) distributions, depending on the estimability of the parameters' magnitude prior to model fitting

$$\mu_\Phi \sim C(1{,}0.5),\ \sigma_\Phi, \mu_n, \sigma_n \sim C(0{,}1) \text{ and } a, b \sim N(0{,}1).$$

Additionally, we enforce $\mu_\Phi, \sigma_\Phi, \sigma_n \geq 0$ and $b \in [-1{,}1]$, to ensure positive scale parameters.

Extraction of gene-wise fitness scores. After fixing the normalization factors and determining the abundance and replicate dependence of the mixing coefficient θ_{rgk} within the ZINB model, we proceeded to fit the ZINB model (Eq 1) to the complete data set to extract the gene-wise log-fold changes logFC_g and the gene-wise dispersion ϕ_g. Again, we improved the variance modeling by imposing a hierarchical Gaussian prior:

$$\phi_g \sim N(\mu_\phi, \sigma_\phi).$$

We set the scale parameter in the prior distribution of log-fold changes to a large value

$$\text{logFC}_g \sim N(\mu_{\text{logFC}}, 5)$$

to prevent undue shrinkage of the effect sizes. Importantly, we chose not to center the prior distribution at zero. In TraDIS screens, it is common for the number of disadvantageous mutations to exceed advantageous ones, leading to a negative mean μ_{logFC}. The hyper parameters followed Cauchy distributions, i.e. $\mu_{\text{logFC}}, \sigma_\phi \sim C(0{,}1)$ and $\mu_\phi \sim C(1{,}0.5)$. Again, we ensured positive values for the parameters μ_ϕ and σ_ϕ. For figures and tables, we converted the model parameters logFC_g to fitness scores ($\log_2\text{FC}_g$) changing from the natural logarithm to base 2.

Fitting the Bayesian models to data from the TraDIS screen. To derive the posterior distributions of the model parameters, we employed the probabilistic programming language Stan[2] (v.2.31.0). The statistical models were fitted to the TraDIS screen data running two chains of 1000 Markov Chain Monte Carlo (MCMC) samples each (method=sample num_samples=1000 num_warmup=1000 adapt delta=0.95 algorithm=hmc engine=nuts max_depth=12).

Determine statistical significance of log-fold changes. Fitting the Bayesian ZINB model with log-fold changes to the TraDIS screen data yields posterior distributions for the gene-wise log-fold changes. The expected gene-wise log-fold change is given by the median. To assess statistical significance and control the false discovery rate (FDR), we computed z-values $z_g = \frac{\text{logFC}_g}{\Delta\text{logFC}_g}$, where ΔlogFC_g is the standard deviation of the posterior distribution. These z-values are expected to follow a normal distribution under the null hypothesis[3]. Indeed, the distribution of the z-values (Extended Data Fig. 3k) closely resembles a standard normal distribution with an excess of negative z-values which correspond to the genes of interest, i.e. potential invasion factors.

Assuming that at least 10% of genes affect fitness in infection (a rather conservative assumption), the FDR for a negative z-value cutoff is determined by

$$\text{FDR}(z) = 2 \cdot \frac{0.9 \cdot \Phi(z|0{,}1)}{|\{z_g | z_g \leq z\}|},$$

where Φ is the cumulative normal distribution and $|\{z_g | z_g \leq z\}|$ the number of genes with $z_g \leq z$. Similarly, the FDR for positive z-values is given by

$$\text{FDR}(z) = 2 \cdot \frac{0.9 \cdot \Phi(-z|0,1)}{|\{z_g|z_g \geq z\}|}.$$

The resulting curve is depicted in Extended Data Fig. 3l.

We explored two alternative methods to assign FDR to the log-fold changes each presenting its own challenges. One approach involves calculating Bayesian p values, representing the fraction of the 2000 MCMC samples s in agreement with the null hypothesis. However, due to the precision limitations inherent in measuring log-fold changes in the experimental setup, the finite interval $[-h_0, h_0]$ is assumed to align with the null hypothesis, resulting in the following Bayesian p values

$$p_g = \begin{cases} \frac{|\{s|s \geq -h_0\}|}{2000}, \text{ if } \text{logFC}_g < 0 \\ \frac{|\{s|s \leq h_0\}|}{2000}, \text{ if } \text{logFC}_g > 0 \end{cases}.$$

This approach poses the challenge of defining the distribution of p values under the null hypothesis, a prerequisite for converting Bayesian p values into FDRs.

Alternatively, a more elegant method involves the use of Bayes' factors[4]. Bayes' factors quantify how much less likely the null hypothesis is compared to the median log-fold change after fitting the model to the data as compared to the prior estimates

$$\text{Bayes' factor}\left(\text{logFC}_g\right) = \frac{\Pr(\text{data}|0)}{\Pr\left(\text{data}\middle|\text{logFC}_g\right)} = \frac{\Pr(0|\text{data})\Pr\left(\text{logFC}_g\right)}{\Pr\left(\text{logFC}_g|\text{data}\right)\Pr(0)}.$$

While a Bayes' factor smaller than 0.1 is considered strong evidence, this is not inherently corrected for multiple hypothesis testing. Considering the challenge associated with defining the null hypothesis of Bayesian p values and correcting Bayes' factors for multiple hypothesis testing, we opted to use z-values to assign FDRs to log-fold changes.

Supplementary Note 2. Caco-2 cells culture and infections.

Caco-2 cells (ATCC HTB-37) were grown in DMEM GlutaMAX (Gibco, #31966–021) supplemented with 10% heat-inactivated fetal bovine serum (FBS; Gibco, #10270106) and 0.1mM Non-Essential Amino Acids (Gibco, #11140035) at 37°C with 10% CO2. Cultures were passaged three times/week with 100IU/ml penicillin and 100µg/ml streptomycin, but antibiotics were omitted during infection experiments. 150,000 Caco-2 cells were seeded in 12-well plates 24h before infection. The indicated *Shigella* strains were grown ON in LB containing appropriate antibiotics at 30°C, diluted 1:50, and subcultured for 2h at 37°C without antibiotics. Bacteria were added to each well and spun down at 300g for 10min. At 1h p.i. the culture medium was replaced with fresh medium containing 200µg/ml gentamicin and the cells further incubated for 1h. For infections extending 3h incubation, media was replaced with 20µg/ml gentamicin media up to 6h p.i.. At 2h and 6h p.i. cells were washed and lysed adding 0.1% Na-deoxycholate, the lysates were diluted and plated on LB agar plates with appropriate antibiotics, followed by enumeration of colony-forming units (CFUs). Number of generations (n) in Extended Data Fig. 1d was calculated according to the formula n = 3.3*Log (N_{6h} / N_{2h}) where N_{6h} and N_{2h} are bacterial intracellular population sizes at 6h and 2h p.i., respectively. Generation time (G) in Extended Data Fig. 1d was calculated according to the formula G = t / n, where t is the time between the two time points (in min) and n is the number of generations.

Supplementary Note 3. Bacterial RNA extraction

Total RNA was extracted with acid phenol. In brief, cultures were spun down and the pellet resuspended in TES buffer (10 mM Tris HCl pH8, 1mM EDTA, 150mM NaCl). SDS (final concentration 1%) was added, and the tubes incubated for 5min at 95°C. Samples were cooled on ice and incubated for 3min at 65°C, before adding 1 volume of acid phenol. The samples were incubated at 65°C for 10min and centrifuged for 10min at 18,000g at 4°C. The aqueous phase was transferred to a clean tube, and 1 volume of chloroform:isoamyl alcohol 24:1 added to each tube. The samples were mixed vigorously, incubated for 5min at 65°C and centrifuged 5min at 18,000g at 4°C. The aqueous phase was transferred to a clean tube and mixed with 2.5 volumes of ice-cold 100% EtOH and incubated for at least 1 hour at −80°C. The samples were centrifuged for 30min at 18,000g at 4°C, and the pellets washed with 70% ice-cold EtOH. After a second centrifugation for 10min (18,000g, 4°C), the pellets were air dried, dissolved in sterile water and RNA quality was checked by agarose gel electrophoresis.

REFERENCES

1. Jenniches, L. ZINB model for TraDIS infection screens.doi:10.5281/zenodo.15096674.
2. Carpenter, B. *et al.* Stan: A Probabilistic Programming Language. *J. Stat. Softw.* **76**, (2017).
3. Efron, B. Microarrays, Empirical Bayes and the Two-Groups Model. *https://doi.org/10.1214/07-STS236* **23**, 1–22 (2008).
4. Jeffreys, H. Theory of Probability. 3rd Edition, Clarendon Press, Oxford. 470 (1961).
