# Supplementary material for: A scalable gut epithelial organoid model reveals the genome-wide colonization landscape of a human-adapted pathogen
Source: Nat Genet. 2025 Jun 12;57(7):1730–41. doi: 10.1038/s41588-025-02218-x (PMC12283395; doi:10.1038/s41588-025-02218-x)
Supplement: Supplementary file 2 — Reporting Summary [file 41588_2025_2218_MOESM2_ESM.pdf]

nature portfolio

Corresponding author(s): Maria Letizia Di Martino, Laura Jenniches, Lars Barquist, Mikael Sellin

Last updated by author(s): Apr 7, 2025

Reporting Summary

Nature Portfolio wishes to improve the reproducibility of the work that we publish. This form provides structure for consistency and transparency in reporting. For further information on Nature Portfolio policies, see our Editorial Policies and the Editorial Policy Checklist.

Statistics

For all statistical analyses, confirm that the following items are present in the figure legend, table legend, main text, or Methods section.

n/a	Confirmed	
☐	☒	The exact sample size (*n*) for each experimental group/condition, given as a discrete number and unit of measurement
☐	☒	A statement on whether measurements were taken from distinct samples or whether the same sample was measured repeatedly
☐	☒	The statistical test(s) used AND whether they are one- or two-sided<br>*Only common tests should be described solely by name; describe more complex techniques in the Methods section.*
☒	☐	A description of all covariates tested
☐	☒	A description of any assumptions or corrections, such as tests of normality and adjustment for multiple comparisons
☐	☒	A full description of the statistical parameters including central tendency (e.g. means) or other basic estimates (e.g. regression coefficient) AND variation (e.g. standard deviation) or associated estimates of uncertainty (e.g. confidence intervals)
☐	☒	For null hypothesis testing, the test statistic (e.g. *F*, *t*, *r*) with confidence intervals, effect sizes, degrees of freedom and *P* value noted<br>*Give P values as exact values whenever suitable.*
☐	☒	For Bayesian analysis, information on the choice of priors and Markov chain Monte Carlo settings
☒	☐	For hierarchical and complex designs, identification of the appropriate level for tests and full reporting of outcomes
☒	☐	Estimates of effect sizes (e.g. Cohen's *d*, Pearson's *r*), indicating how they were calculated

Our web collection on statistics for biologists contains articles on many of the points above.

Software and code

Policy information about availability of computer code

Data collection	No software was used for data collection.
Data analysis	- For TraDIS under different growth conditions we used the Bio-TraDIS pipeline (v1.4.5) to align and quantify reads and EdgeR (v4.0.1) to analyze significant changes in fitness.<br>- For differential gene expression we used STAR (v2.6.0a) and EdgeR (v4.0.1) (Robinson et al 2010).<br>- For the main TraDIS screen in basal-out enteroid infections we used a custom Bayesian ZINB model, available through the following Zenodo link: https://doi.org/10.5281/zenodo.15096674.<br>- For Mass spectrometry-based proteomics MSFragger (v3.0) was used (Kong et al, 2017). Data was normalized using vsn (v3.74.0) (Huber et al, 2002) and statistical significance was determined using limma (v3.62.2) (Ritchie et al, 2015).<br>- For codon usage analysis we used a Python (3.9.16) script and the BioPython (1.81) library. All code is open source and available at https://github.com/Oftatkofta/codon_counter and through the following Zenodo link: https://zenodo.org/records/15100662.

For manuscripts utilizing custom algorithms or software that are central to the research but not yet described in published literature, software must be made available to editors and reviewers. We strongly encourage code deposition in a community repository (e.g. GitHub). See the Nature Portfolio guidelines for submitting code & software for further information.

Data

Policy information about availability of data

All manuscripts must include a data availability statement. This statement should provide the following information, where applicable:

- Accession codes, unique identifiers, or web links for publicly available datasets
- A description of any restrictions on data availability
- For clinical datasets or third party data, please ensure that the statement adheres to our policy

- Transcriptomic data and TraDIS data have been deposited in the Gene Expression Omnibus (GEO) database, with the SuperSeries no. GSE267520, link: https://www.ncbi.nlm.nih.gov/geo/query/acc.cgi?acc=GSE267520.

- The mass spectrometry proteomics data have been deposited at the ProteomeXchange Consortium via the PRIDE partner repository with the dataset identifier PXD046629, link: https://www.ebi.ac.uk/pride/archive/projects/PXD046629.

Research involving human participants, their data, or biological material

Policy information about studies with human participants or human data. See also policy information about sex, gender (identity/presentation), and sexual orientation and race, ethnicity and racism.

Reporting on sex and gender	Not relevant to our study.
Reporting on race, ethnicity, or other socially relevant groupings	Not relevant to our study.
Population characteristics	Not relevant to our study. No data on population characteristics was collected/used.
Recruitment	Human adult stem cell-derived enteroids/colonoids were established from jejunal tissue re-sected during bariatric surgery (enteroids), or from morphologically normal non-tumor colon tissue resected during elective colon cancer surgery (colonoids), in all cases following the subject's informed consent. To ensure the anonymity of tissue donors, samples were pseudonymized. Patients' identities were not accessible to laboratory personnel.
Ethics oversight	The procedures were approved by the local governing body (Etikprövningsmyndigheten, Sweden, Sweden) under license nr 2010-157 with addenda 2010-157-1 and 2020-05754, and license nr 2023-01524-01.

Note that full information on the approval of the study protocol must also be provided in the manuscript.

Field-specific reporting

Please select the one below that is the best fit for your research. If you are not sure, read the appropriate sections before making your selection.

☒ Life sciences ☐ Behavioural & social sciences ☐ Ecological, evolutionary & environmental sciences

For a reference copy of the document with all sections, see nature.com/documents/nr-reporting-summary-flat.pdf

Life sciences study design

All studies must disclose on these points even when the disclosure is negative.

Sample size	- TraDIS experiments under different growth conditions were done in biological triplicates.<br>- TraDIS pilot experiments in basal-out enteroids were done in biological triplicates.<br>- RNA-seq experiments were done in biological triplicates.<br>- Main TraDIS screen in basal-out enteroid infections was done using 43 biological replicate sub-libraries. Sample size was predetermined experimentally ans in silico as detailed in the methods (see "TraDIS pilot screen in basal-out enteroids" and "In silico determination of number of replicates").<br>- Proteomic profiling was done using four biological replicates per strain.<br>- Barcoded assays were performed at least in duplicates (In this setup each replicate employed two biological replicates for each genotype, allowing for a powerful and internally controlled comparison).<br>- All other experiments were performed with at least 3 biological independent replicates. Exact sample sizes are indicated in each figure legend.<br>Our replicate numbers were consistent with those reported in similar studies within the field, indicating that our experimental design was appropriately aligned with established standards. Additionally, the validation of the TraDIS results through multiple approaches, along with the high reproducibility of all experiments, indicates that our sample sizes were sufficient.
Data exclusions	For the main TraDIS screen in basal-out enteroid infections, sub-library 11 was excluded due to low sequencing read counts.
Replication	Experimental replicates are indicated in the figure legends, and each experiment was repeated at least two times.

Randomization	Not relevant to our study since all bacterial strains/samples were treated in the same way.
Blinding	Not relevant to our study since all bacterial strains/samples were treated in the same way.

Reporting for specific materials, systems and methods

We require information from authors about some types of materials, experimental systems and methods used in many studies. Here, indicate whether each material, system or method listed is relevant to your study. If you are not sure if a list item applies to your research, read the appropriate section before selecting a response.

Materials & experimental systems

n/a	Involved in the study
☐	☒ Antibodies
☐	☒ Eukaryotic cell lines
☒	☐ Palaeontology and archaeology
☒	☐ Animals and other organisms
☒	☐ Clinical data
☒	☐ Dual use research of concern
☒	☐ Plants

Methods

n/a	Involved in the study
☒	☐ ChIP-seq
☒	☐ Flow cytometry
☒	☐ MRI-based neuroimaging

Antibodies

Antibodies used	Anti-FLAG antibody (Sigma, #F1804) for Western Blots.
Validation	Anti-FLAG antibody has been validated by Sigma, as specified in the product description, accessible through the homepage of the manufacturer. From Sigma: "We have employed an affinity resin to purify an ANTI-FLAG M2 monoclonal antibody exhibiting excellent specificity and high sensitivity. This affinity-purified ANTI-FLAG M2 antibody has been utilized to detect tagged fusion proteins in multiple expression systems, displaying virtually exclusive selectivity for the target protein band in Western blot immunostaining."<br>To further confirm the selectivity of the antibody in our system, we included a negative control sample which does not express the FLAG-tagged protein of interest, in all western blots.

Eukaryotic cell lines

Policy information about cell lines and Sex and Gender in Research

Cell line source(s)	- Human adult stem cell-derived enteroids/colonoids were established from jejunal tissue resected during bariatric surgery (enteroids), or from morphologically normal non-tumor colon tissue resected during elective colon cancer surgery (colonoids), in all cases following the subject's informed consent.<br>- Caco-2 cells (ATCC HTB-37)
Authentication	Eurofins authenticated Caco-2 cells using STR profiling. Caco-2 cells were continuously monitored for their well-defined phenotypic behavior including capacity to form a high-TEER monolayer and microvilliated cell morphology atop PET transwell inserts.
Mycoplasma contamination	- Enteroids and Colonoid cultures were not tested for mycoplasma contamination. Sentinel cultures from the cell culture facility were however tested for Mycoplasma contamination and were found to be negative during the entire duration of this study.<br>- Caco-2 cells were confirmed to be mycoplasma-free by PCR following growth in culture medium without antibiotics.
Commonly misidentified lines<br>(See ICLAC register)	No commonly misidentified cell lines were used in the study.

Plants

Seed stocks	*Report on the source of all seed stocks or other plant material used. If applicable, state the seed stock centre and catalogue number. If plant specimens were collected from the field, describe the collection location, date and sampling procedures.*
Novel plant genotypes	*Describe the methods by which all novel plant genotypes were produced. This includes those generated by transgenic approaches, gene editing, chemical/radiation-based mutagenesis and hybridization. For transgenic lines, describe the transformation method, the number of independent lines analyzed and the generation upon which experiments were performed. For gene-edited lines, describe the editor used, the endogenous sequence targeted for editing, the targeting guide RNA sequence (if applicable) and how the editor was applied.*
Authentication	*Describe any authentication procedures for each seed stock used or novel genotype generated. Describe any experiments used to assess the effect of a mutation and, where applicable, how potential secondary effects (e.g. second site T-DNA insertions, mosiacism, off-target gene editing) were examined.*
