# Supplementary material for: A scalable gut epithelial organoid model reveals the genome-wide colonization landscape of a human-adapted pathogen
Source: Nat Genet. 2025 Jun 12;57(7):1730–41. doi: 10.1038/s41588-025-02218-x (PMC12283395; doi:10.1038/s41588-025-02218-x)

Figure 4j - Left Panel (*mnmE* complementation)
Representative Western Blot

Blot
IpaA - FlagTag Antibody

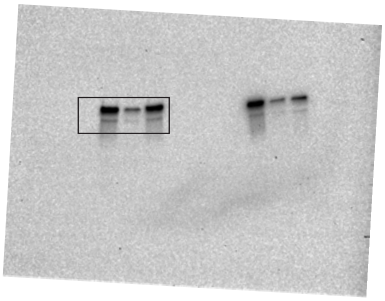

Gel
Total Proteins - Stain Free

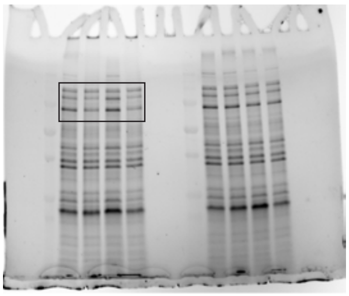

Merged image: Blot + Ladder

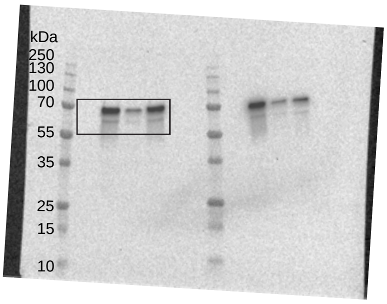

Figure 4j - Right Panel (*mnmG* complementation)
Representative Western Blot

Blot
IpaA - FlagTag Antibody

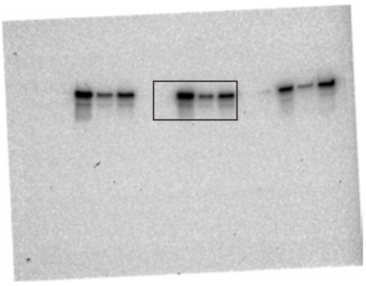

Gel
Total Proteins - Stain Free

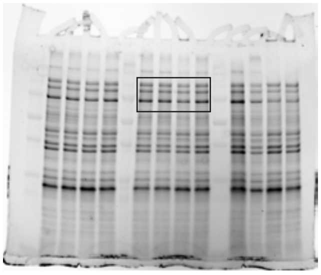

Merged image: Blot + Ladder

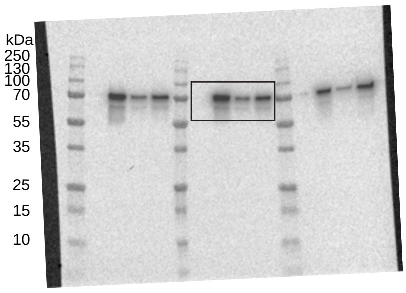

Extended Data Figure 7a - Quantitative Western Blots (*mnmE* complementation)

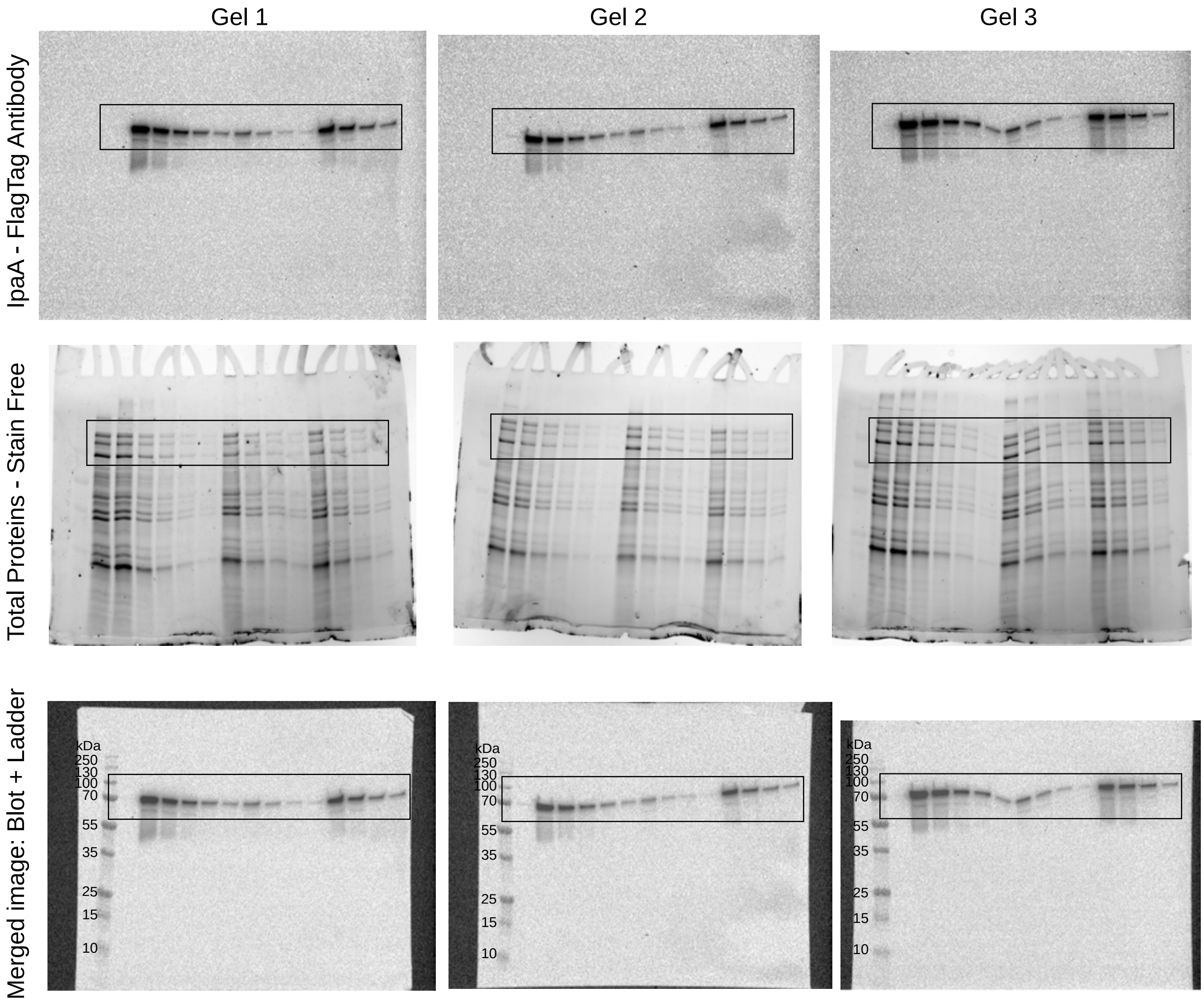

Extended Data Figure 7b - Quantitative Western Blots (*mnmG* complementation)

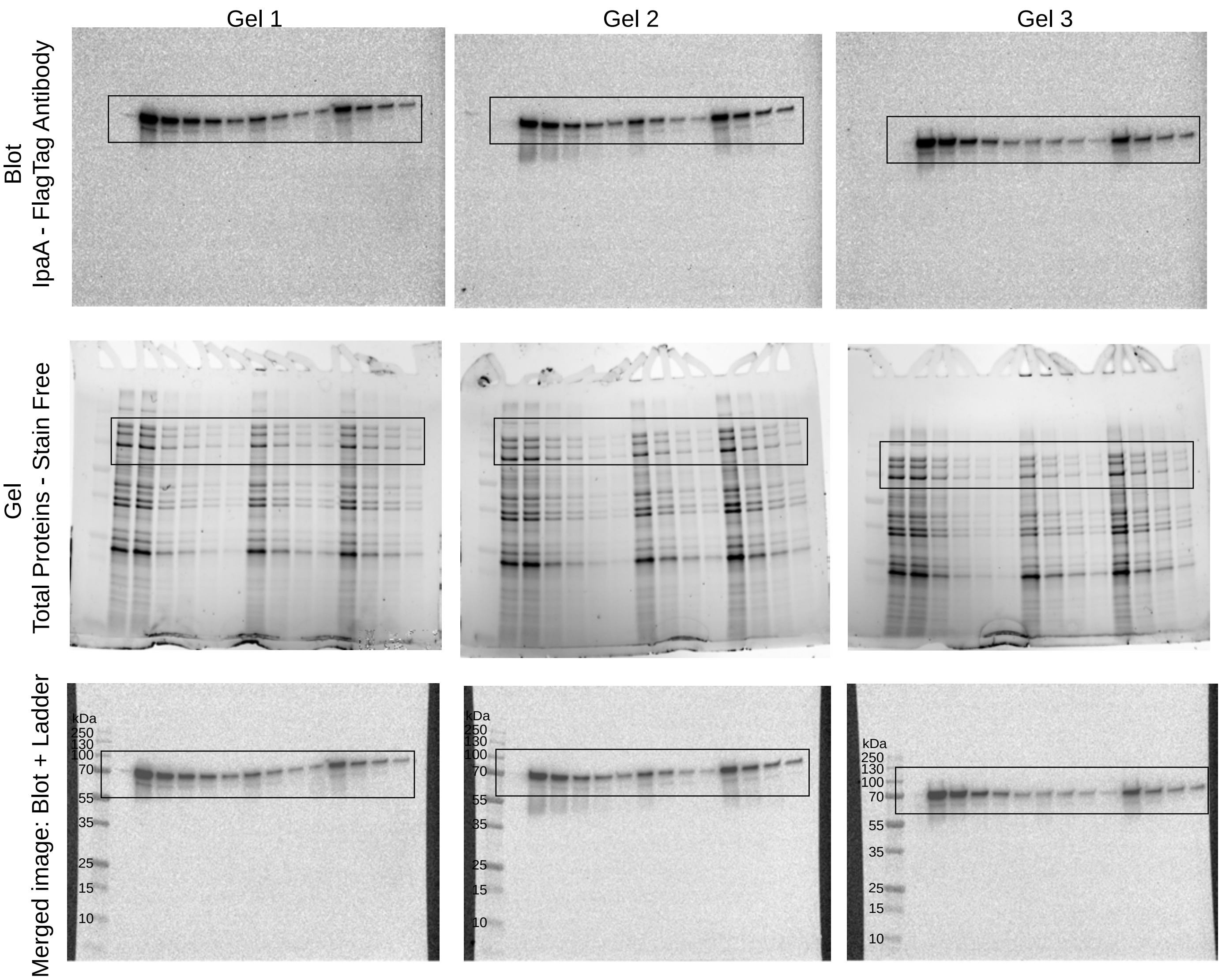

Supplement: Supplementary file 13 — Unprocessed western blots/gels. [file 41588_2025_2218_MOESM13_ESM.pdf]
